# CREB5 hypermethylation involved in the ganglioside GM1 therapy of Parkinson’s disease

**DOI:** 10.3389/fnagi.2023.1122647

**Published:** 2023-05-31

**Authors:** Rui Wang, Shanshan Tong, Mengdi Wang, Junjie Zou, Nan Wang, Fengjiao Sun, Xiaosheng Zhou, Jinbo Chen, Hongcai Wang

**Affiliations:** ^1^Department of Neurology, Binzhou Medical University Hospital, Binzhou, Shandong, China; ^2^Department of Neurology, Penglai People’s Hospital, Yantai, China; ^3^Medical Research Center, Binzhou Medical University Hospital, Binzhou, Shandong, China

**Keywords:** monosialotetrahexosylganglioside (GM1), Parkinson’s disease (PD), DNA methylation, rotenone (ROT), cell apoptosis

## Abstract

**Introduction:**

The treatment with monosialotetrahexosylganglioside (GM1) improves the symptoms of Parkinson’s disease (PD). The alteration of DNA methylation in the blood was examined to investigate epigenetic modification by GM1 treatment.

**Methods:**

After a 28-day continuous intravenous infusion of GM1 (100mg), the motor and non-motor symptoms were evaluated by UPDRS III, Mini-mental state examination (MMSE) scores, FS-14, SCOPA-AUT, and PDQ-8. Moreover, blood samples were collected and PBMC was isolated. Genome-wide DNA methylation was performed by an 850K BeadChip. RNA levels and apoptosis were examined by RT-PCR and flow cytometry in rotenone-based cell models. The CREB5 plasmid was transfected by electroporation into SH-SY5Y cells. We also identified 235 methylation variable positions achieving genome-wide significance in 717558 differentially methylated positions (DMPs) (*P* = 0.0003) in comparison of pre-treatment with post-treatment measurements (statistical analysis paired-samples *t*-test).

**Results:**

By searching the Gene Expression Omnibus (GEO) dataset and GWAS, 23 methylation variable positions were screened. Moreover, there are 7 hypomethylated methylation variable positions correlated with the scores of motor symptoms (UPDRS III scale). According to KEGG pathways enrichment analysis, the methylated genes CACNA1B (hypomethylated), CREB5 (hypermethylated), GNB4 (hypomethylated), and PPP2R5A (hypomethylated) were enriched in the dopaminergic synapse pathway. Pretreated with GM1 (80 μM) for 1 h, cell apoptosis and impaired neurite outgrowth were inhibited in rotenone-induced PD cell models. The RNA expression of CREB5 was increased in rotenone-treated SH-SY5Y cells. GM1 treatment decreased rotenone-induced CREB5 gene expression. The enhancement of CREB5 gene expression suppressed the protective role of GM1 in rotenone-induced cell apoptosis.

**Discussion:**

The application of GM1 improves the motor and non-motor symptoms of PD associated with the decreased CREB5 expression and the hypermethylation of CREB5.

**Clinical trial registration:**

https://www.chictr.org.cn/showproj.html?proj=120582t, identifier ChiCTR2100042537.

## 1. Introduction

Parkinson’s disease (PD) is among the most prevalent neurodegenerative disorders. It had been reported that PD affected 1% of the population above 60 years in the world, and men had a significantly greater incidence and a slightly earlier onset of PD than women (Twelves et al., 2003; Pringsheim et al., 2014; Tysnes and Storstein, 2017). At present, the therapy for Parkinson’s disease (PD) is mainly done through levodopa replacement and cannot delay the progress of PD (Bogetofte et al., 2020). Although improvement for many of the motor symptoms of the disease can be obtained with available pharmacotherapies, functional ability continues to deteriorate over time (Schneider et al., 2015). Therefore, the development of disease-modifying therapies is an area of intense interest. Some studies indicated that the intravenously administered monosialotetrahexosylganglioside (GM1), which crosses the human blood-brain barrier, played a neuroprotective role in the pathogenesis of PD (Chiricozzi et al., 2020). Moreover, GM1 was a glycosphingolipid that was normally found in the brain and was a normal part of the outer covering or membrane of nerve cells (Aureli et al., 2016). The application of GM1 did not only improve the symptoms but also had a protective effect, including the promotion of synaptic growth of nerve cells and neuronal differentiation (Ledeen and Wu, 2015).

Aberrant epigenetic modifications were involved in the pathogenesis of a range of diseases and were non-invasively detectable in blood as diagnostic biomarkers of disease (Henderson et al., 2021). DNA methylation, which is an epigenetic modification of DNA, played an important role in transcriptional regulation in the chromatin (Wüllner et al., 2016). Furthermore, DNA methylation most likely documented complex environment-gene interactions and regulated drug effects and side effects (Reynolds, 2014).

The deficiency of GM1 was certificated in the substantia nigra and occipital cortex in the pathology of PD. It was also assumed that GM1 suppressive factors were potential risk factors for PD, and GM1 replacement was thought to be a disease-altering therapy (Ledeen et al., 2022). Another study showed that long-term GM1 application by PD patients was safe and produced some clinical benefit for PD patients (Magistretti et al., 2019). GM1 therapy enhanced epigenetic activation in neuronal cells (Tsai et al., 2016).

There is a growing interest in exploring the modified role of DNA methylation in PD pathology. The previous study indicated that the status of PD patients might be associated with changes in DNA methylation levels in blood (Chuang et al., 2017). In our study, after treatment with GM1, DNA methylation was investigated by isolating PBMC from the blood sample. The purposes of the study were: (1) to study whether PD status is associated with DNA methylation levels in blood; (2) to characterize genome-wide significant markers in terms of biological pathways.

## 2. Materials and methods

### 2.1. Participants

This study (ChiCTR2100042537) was conducted at two sites in the Binzhou Medical University Hospital China and Yantai Penglai People’s Hospital between March 2021 and July 2021. This study was approved by the local Institutional Committee for Protection of Human Subjects, and informed consent was obtained from all subjects and their legal guardians prior to commencement of the study.

Subjects were men or women between 30 and 70 years of age with a diagnosis of idiopathic PD consistent with the UK PD Society brain bank PD diagnostic criteria. Inclusion criteria included “off” UPDRS motor score between 10 and 40, “off” Hoehn and Yahr staging of 1.0 to 3.0, Mini Mental State Exam (MMSE) score of ≥25. The subjects who received a stable dose of Madopar, dopamine receptor agonists or monoamine oxidase type B inhibitors for 1 month before study enrollment. Main exclusion criteria included: abrupt onset Parkinsonism; history of Guillain-Barré syndrome; use of anti-coagulant therapy; history of brain surgery for PD; patients who used GM1 within 3 months; Patients with positive ganglioside antibody; patients allergic to GM1 injection. Research on patients requires the supervision of a competent and appropriately qualified physician. Research must conform to generally accepted scientific principles. The physician must fully inform the subjects about which aspects of their care are related to the research. Every precaution must be taken to protect the privacy of research subjects and the confidentiality of their personal information. The refusal of a patient to participate in a study or the patient’s decision to withdraw from the study must never adversely affect the patient-physician relationship.

### 2.2. Drug administration

Ganglioside GM1 [national medicine permission number (NMPN) H20051486], extracted from bovine brain, was obtained from Qilu Pharmaceutical Corporation (Jinan, China). One hundred mg of Ganglioside-Monosialic Acid in 250 ml of 0.9% sodium chloride was given intravenously daily to each patient for 28 consecutive days. The blood sample was collected from patients with GM1 pre- and post-treatment, and then peripheral blood mononuclear cells (PBMC) were isolated.

### 2.3. DNA isolation and infinium human methylation 850K BeadChip

Peripheral blood samples were collected from 6 PD patients, and mononuclear cells were separated through Ficoll-Paque (GE Heatlthcare) gradient centrifugation. The total DNA was prepared by proteinase K/phenol method. DNA isolation and genome-wide DNA methylation patterns were analyzed by Infinium Human Methylation 850 K BeadChips (Illumina), which determine the methylation levels of 853,307 CPG sites. After performing bisulfite treatment with the EZ DNA Methylation Kit (Zymo Research) following the manufacturer’s procedure, processed DNA samples were then hybridized to the BeadChip (Illumina) according to the Illumina Infinium HD Methylation Protocol. Illumina intensity data (IDAT) files from the chip were further processed by the R/Bioconductor (version 3.3.3) package ChAMP. DNA methylation level was reported as β-value, ranging from zero to one, where zero represents a non-methylated probe and one represents a fully methylated probe, for every CPG site. Probes that had a detection *P*-value of >0.01 and those located on the X and Y chromosomes were filtered away. We also removed SNP-related probes and all multi-hit probes. BMIQ (Beta MIxture Quantile dilation) algorithm was used to correct for the Infinium type I and type II probe bias.

Furthermore, we identified differently methylated probes (DMPs) at a significance level of a Benjamini-Hochberg adjusted *P* < 0.05 and performed gene set enrichment analysis to determine whether genes involved in these significant DMPs are enriched for specific biological terms or pathways. Differentially methylated regions (DMRs) were identified by Bumphunter with default settings.

### 2.4. Cell culture

Acquired from the American Type Culture Collection (ATCC, USA), human neuroblastoma SH-SY5Y cells were cultured indefinitely at 37°C in a humidified 5% CO_2_ incubator with a DMEM-F12 ratio of 1:1 (HyClone) containing 10% heat-inactivated fetal bovine serum (GIBCO-BRL Life Technology).

### 2.5. Plasmid construction and DNA transfection by electroporation

N-terminal 3xFLAG-tagged CREB5 (Supplementary Table 1) was constructed by high-fidelity PCR and cloned into pEnCMV vectors from the MiaoLing Plasmid Sharing Platform (Wuhan, China). The CREB5 plasmid was transfected into SH-SY5Y cells using cell electroporation by Celetrix Page (Manassas, VA 20109 US). SH-SY5Y cells were harvested into 15-ml centrifuge tubes and spun down at 1000 rpm for 5 min. The cells were gently resuspended in electroporation buffer. Add 2 μg of DNA and make sure the total volume is 20 μl. After locking the electroporation tube into the adapter, cell line models (530 v) were chosen on the screen, and electroporation was initiated. Upon appearance of an oscillograph indicating electroporation was complete, the sample was removed, and suspended cells were transferred to an antibiotic-free culture medium. Fresh culture medium was renewed after 24 h when the cells were at high plating density.

### 2.6. Apoptosis assay

The SH-SY5Y cells were treated with rotenone (ROT) (2 μM) for 36 h after transfecting pEnCMV-CREB5-3xFLAG for 48 h. The cells were harvested into 1.5-ml centrifuge tubes and spun down at 500 g for 5 min. With cold PBS, the cells were washed two times, and a volume of 100 μl of binding buffer (Apoptosis Detection Kit, Meilunbio. Co., Ltd.) was added into the tube. A volume of 5 μl Annexin-V FITC and 5 μl PI solutions were added into the tube and incubated in the dark for 15 min. Each tube was then filled with 400 μl of 1× binding buffer and vortexed gently before being analyzed with a flow cytometer. Approximately 10,000 occurrences were noted based on viable, early apoptotic and late apoptotic, and necrotic cells.

### 2.7. RNA isolation and quantitative polymerase chain reaction (qPCR)

Reverse transcription qPCR was used to quantify the amount of CREB5 mRNA using an ABI ViiA7 instrument (Thermo Fisher Scientific). Total RNA from SH-SY5Y cells was isolated using the RNeasy Mini kit (Qiagen) according to the manufacturer’s instructions. Single-stranded cDNA was created with reverse transcription using a first-strand cDNA synthesis kit and random primers. All cDNA samples for the subsequent qPCR analyses were stored at −20°C. The 2^–ΔΔCt^ technique was used to determine the relative level of mRNA of the target gene. 18s rRNA was used as the housekeeping gene. The primers were designed to target CREB5 and had the following sequences: Forward: 5′- CTTGCTCCCAGCAACAAGTCA-3′, Reverse: 5′-GCTGAGGGTGGAGCTTCCTA-3′.

### 2.8. Statistics analysis

Paired samples *t*-test were used to compare pre- and post-treatment scores. Blood components were assessed by paired samples *t*-test and the Wilcoxon Signed-Rank Test. Pearson’s r measures the linear relationship between UPDRSIII and the levels of GMP. Apoptotic cells, RNA expression, and the quantification of neurite outgrowth were assessed by one-way ANOVA. All data were tested for normality using Shapiro–Wilks test and all analysis was performed in IBM SPSS statistics 26 and GraphPad Prism 9. It was considered to be statistically significant when two-sided *p*-value < 0.05.

## 3. Results

### 3.1. The alteration of blood components in GM1 treatment

After continuous intravenous infusion of GM1 for 28 days, PBMCs from pre- and post-treated PD patients (*N* = 21) were statistically analyzed. The results indicated that there was no significant difference in the cell numbers of WBC, RBC, and PLT or the rates of NEUT, LYMPH, and MONO between pre- and post-treatment (Table 1). After GM1 treatment, the BUN (blood urea nitrogen), LDL (low-density lipoprotein), IgG, IgA, IgM, and ALP (alkaline phosphatase) were not obviously changed, but the UA (uric acid) was evidently decreased compared with pretreatment (Table 2).

**TABLE 1:** Compare the cell numbers of WBC, RBC, and PLT and the rates of NEUT, LYMPH, and MONO between pre- and post-treatment.

		*N*	Means ± SD	*t*	df	*P*-value
**Paired samples test**						
Pair 1	WBC pre – WBC post	21	−0.52 ± 1.53	−1.553	20	0.136
Pair 2	RBC pre – RBC post	21	−0.09 ± 0.31	−1.325	20	0.200
Pair 3	PLT pre – PLT post	21	−0.14 ± 32.24	−0.020	20	0.984
Pair 4	NEUT% pre – NEUT% post	21	4.15 ± 11.03	1.723	20	0.100
Pair 5	LYMPH% pre – LYMPH% post	21	−3.22 ± 10.05	−1.468	20	0.158
Pair 6	MONO% pre – MONO% post	21	−0.75 ± 1.65	−2.085	20	0.050

*t*, the test statistic for paired *t*-test; df, the degree of freedom.

**TABLE 2:** Compare the levels of UA (uric acid), BUN (blood urea nitrogen), LDL (low-density lipoprotein), IgG, IgA, IgM, and ALP (alkaline phosphatase) between pre- and post-treatment with GM1.

	Paired differences				
	*N*	Means ± SD	t/z	df	*P*-value
UApre – UApost	21	24.7 ± 37.52	3.018	20	0.007**
BUNpre – BUNpost	21	0.21 ± 0.91	1.047	20	0.296
LDLpre – LDLpost	21	0.01 ± 0.94	0.72	20	0.944
IgGpre – IgGpost	21	3.44 ± 107.56	0.146	20	0.885
IgApre – IgApost	21	2.19 ± 28.80	−0.191	20	0.848
IgMpre – IgMpost	21	−2.18 ± 8.41	−1.185	20	0.250
ALPpre – ALPpost	21	1.94 ± 11.48	0.774	20	0.448

*t*, the test statistic for paired *t*-test; z, the test statistic for Wilcoxon Signed-Rank test; df, the degree of freedom. **p < 0.01.

### 3.2. The application of GM1 improves the motor and non-motor symptoms of PD

**FIGURE 1:**
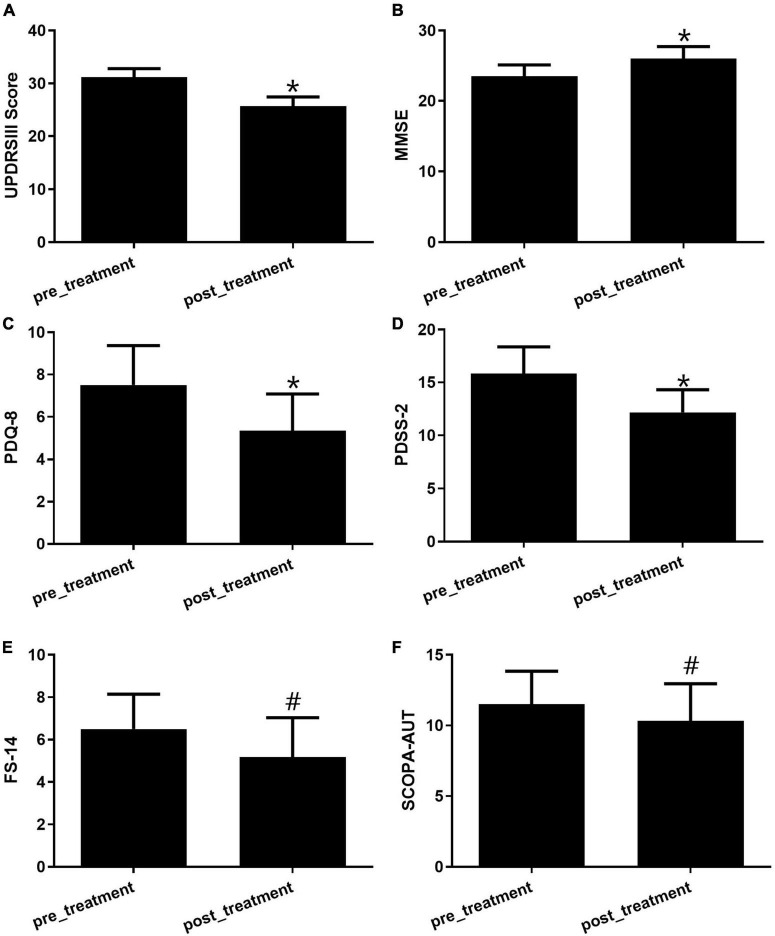
The GM1 treatment improved both motor and non-motor symptoms. **(A)** Motor symptoms were evaluated by the UPDRS III scale, *compared with pretreatment, *P* < 0.05; **(B)** the alteration of cognitive function was examined by the MMSE score, *compared with pretreatment, *P* < 0.05; **(C)** the quality of life was tested by PDQ-8 scale, *compared with pretreatment, *P* < 0.05; **(D)** the evaluation of sleep was used by the PDSS-2, *compared with pretreatment, *P* < 0.05; **(E)** FS-14 is a 14-item self-rating scale for fatigue evaluation, ^#^compared with pretreatment, *P* > 0.05; **(F)** SCOPA-AUT was a well-designed scale for assessing the autonomic dysfunctions of PD patients, ^#^compared with pretreatment, *P* > 0.05.

After treatment with GM1 for 28 days, the motor and non-motor symptoms of PD patients (*N* = 21) altered significantly compared with the symptoms in the pretreatment group. The appropriate scales for changes in motor and non-motor symptoms of PD patients was applied (*n* = 6). For motor symptoms, the subtype frequency changes and the sum of each motor symptom on the UPDRS Part III scale were analyzed statistically between the pre- and post-treatment groups (Figure 1A). Due to intravenous GM1 administration, the total UPDRS III scores apparently decreased (Figure 1A). The MMSE scale, used to measure thinking ability or cognitive impairment, increased significantly in the GM1-treatment group compared to the pre-treatment group (Figure 1B). The Parkinson’s Disease Questionaire-8 (PDQ-8) scale, used to measure quality of life, and the Parkinson’s Disease Sleep Scale-2 (PDSS-2), used to evaluate sleep disturbances, decreased obviously in the GM1-treatment group (Figures 1C, D). There was no significant difference in Fatigue Scale-14 (FS-14) and Scales for Outcomes in Parkinson’s Disease-Autonomic Dysfunction (SCOPA-AUT) between pre- and post-treatment (Figures 1E, F).

### 3.3. Differential distribution of CPG sites and CPG islands (CGI) between the pre- and post-treatment use of GM1

**FIGURE 2:**
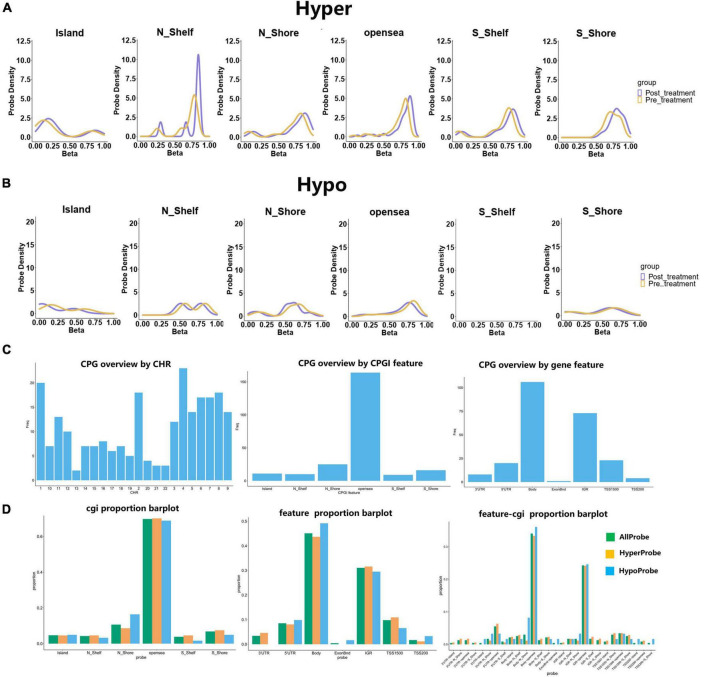
Hyper- and hypo-methylation are distributed in different CPG regions, and the CPG overview and cgi proportion were invested. **(A)** The hypermethylation located in Island, N_shelf, N_shore, opensea, S_Shelf and S_shore. **(B)** The distribution of hypomethylation was analyzed in the pre- and post-treatment groups. **(C)** The CPG overview by CHR, CPGI feature and gene feature. **(D)** The proportion of cgi distributed in different regions. The barplot with different colors represented as all probe, hyperprobe, and hypoprobe.

The distribution of DNA methylation β-values in the hypermethylation was found in various locations, including the island, the N_shelf, the N_shore, the opensea, the S_Shelf, and the S_shore. The distribution of hypomethylation β-values in N_shelf, N_shore, opensea, S_Shelf, S_shore and Island was consistent alteration between pre- and post-treatment groups (Figures 2A, B). CPG overviews by CHR, CPGI features, and gene features were evaluated. Almost 60% of the CGI is located in opensea, 40% in the gene body and 30% in the IGR, 30% in gene body-opensea and 20% in IGR-opensea (Figures 2C, D).

### 3.4. Differential alteration of DNA methylation is correlated with motor symptoms

**FIGURE 3:**
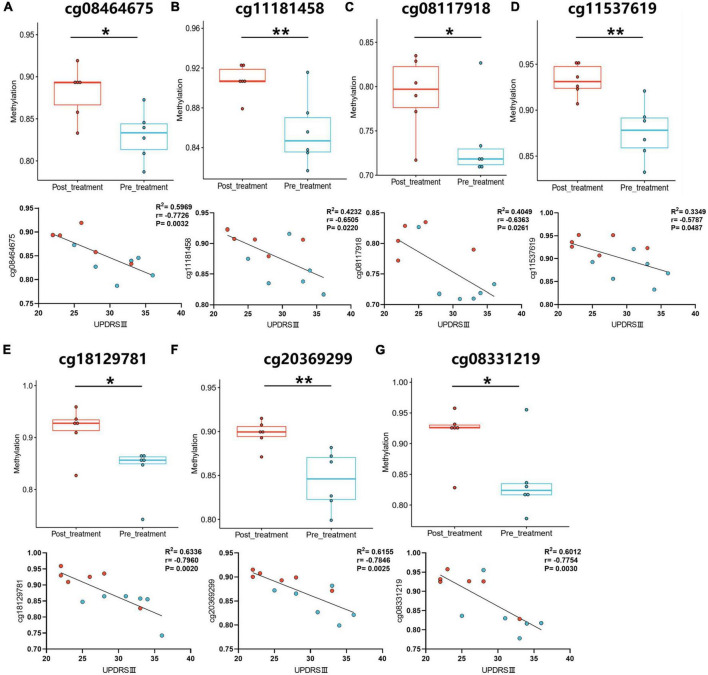
The correlation analysis between UPDRS III motor scale and DMP levels. **(A–G)** The DMP levels were analyzed in pre- and post-treatment groups. The correlation analysis was performed between pre- and post-treatment with GM1. Blue-green dots, pre-treatment groups. Red dots, post-treatment groups. *P < 0.05; **P < 0.01.

After comparing DNA methylation probes in the Gene Expression Omnibus (GEO) database^1^ and GWAS, 23 variable DMPs were screened by comparing measurements before and after treatment (Supplementary Table 2), and a correlation analysis was performed. Pearson’s r measures the linear relationship between DMP levels and the UPDRS III scale. There was statistically significant correlation in 7 of the DMP groups, including cg11537619, cg11181458, cg20369299, cg08464675, cg18129781, cg08331219, and cg08117918 (Figure 3 and Supplementary Table 3).

### 3.5. GM1 administration induced the alteration of DNA methylation patterns in peripheral blood

**FIGURE 4:**
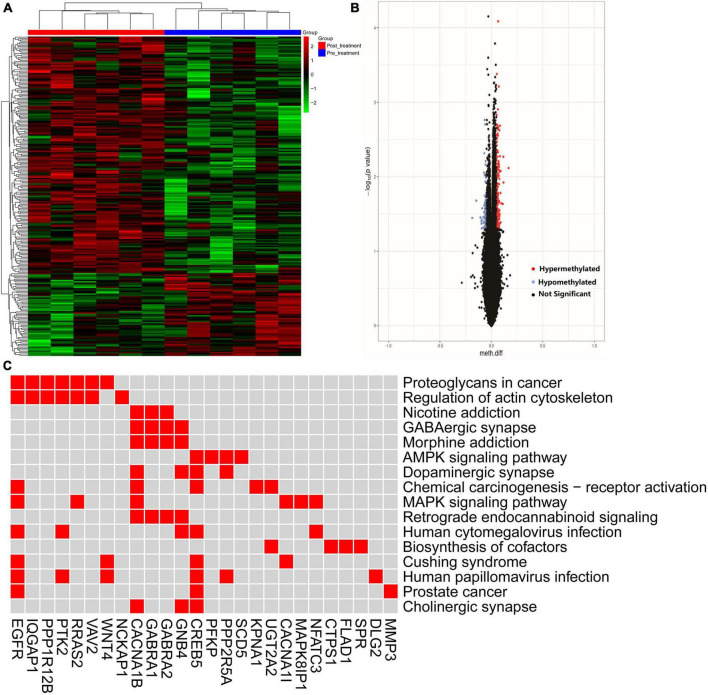
The hyper- and hypo-methylation was depicted by heatmap and volcano. **(A)** The distribution of hyper- and hypo-methylation in pre- and post-treatment groups was revealed in the heatmap plot; **(B)** volcano map was used to show the distribution of differential DNA methylation. Red: hypermethylation, blue: hypomethylation, black: not significant probe. Horizontalcoordinate: Meth. Diff; Verticalcoordinate: –log10 (*p* value). **(C)** Difference methylated genes enriched in the pathway.

The alteration of DNA methylation was analyzed in patients with and without treatment. We identified 235 DMPs characterized by an experiment-wide significant difference in the total of 717558 DMPs between the pre-treatment and post-treatment with GM1 (*P* = 0.0003). Heatmap and volcano plot for differential DNA hypermethylation and hypomethylation were shown in the Figures 4A, B.

The pathway was invested by KEGG analysis of genes with differential DNA methylation. The results revealed that GMP genes were involved in the top 10 pathways including Proteoglycans in cancer, Regulation of actin cytoskeleton, MAPK signaling pathway, Chemical carcinogenesis-receptor activation, GABAergic synapse, Morphine addiction, AMPK signaling pathway, Dopaminergic synapse, Retrograde endocannabinoid signaling and Nicotine addiction. CACNA1B/CREB5/GNB4/PPP2R5A genes enriched in the dopaminergic synapse (Figure 4C).

### 3.6. GM1 prevented rotenone (ROT)-induced cell apoptosis by inhibiting cAMP responsive element binding protein 5 (CREB5) expression

**FIGURE 5:**
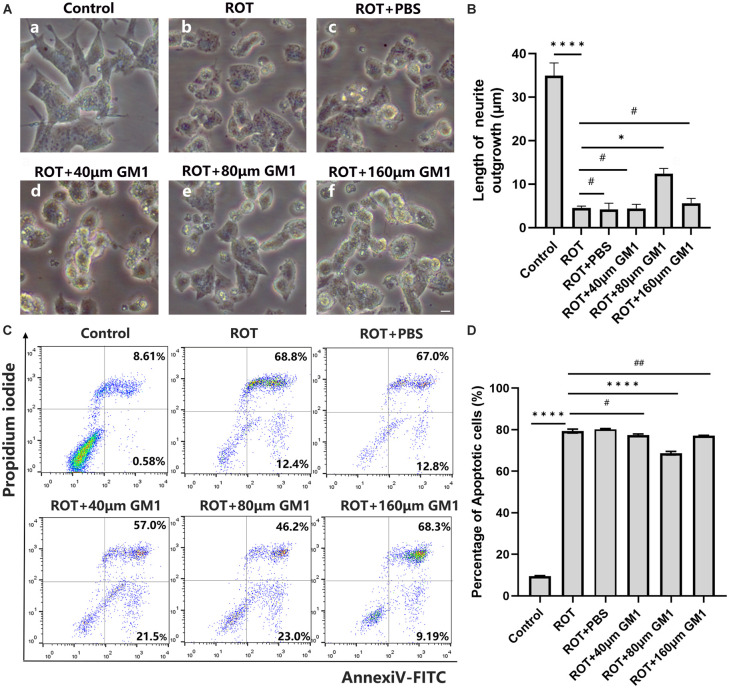
GM1 prevented ROT-induced cell apoptosis. **(A,B)** Impaired neurite outgrowth was inhibited by GM1 addition. Scale bar = 10 μm. **(C,D)** GM1 decreased ROT-induced cell apoptosis. *P < 0.05; ^****^P < 0.0001; ^#,##^*P* > 0.05.

**FIGURE 6:**
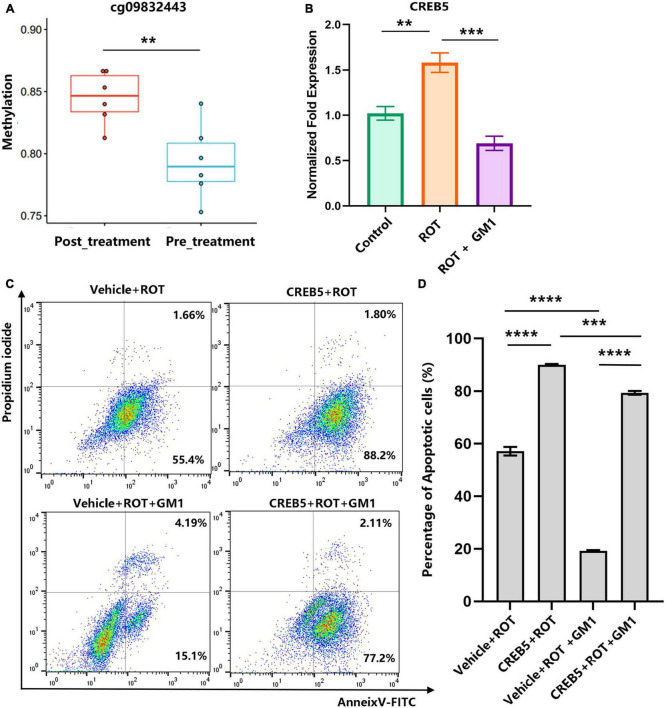
Overexpression of the CREB5 gene inhibited the effects of anti-apoptosis in GM1. **(A)** The DMP levels of the CREB5 gene were analyzed in pre- and post-treatment groups. **(B)** ROT-induced CREB5 elevated relative expression was reduced by GM1. **(C,D)** CREB5-induced cell apoptosis was inhibited by GM1 addition. **P < 0.01; ***P < 0.001; ****P < 0.0001.

In dopaminergic SH-SY5Y cells, pretreated with GM1 (80 μM) for 1h, ROT-induced impaired neurite outgrowth was inhibited obviously (Figures 5A, B). Moreover, cell apoptosis induced by ROT was inhibited obviously due to GM1 treatment (Figures 5C, D). Meanwhile, GM1 treatment induced CREB5 gene hypermethylation compared to pretreatment in clinical therapy (Figure 6A). The CREB5 gene expression was analyzed in ROT-induced SH-SY5Y cell injuries. and the CREB5 gene expression were upregulated in the ROT group compared with the control group. After treatment with GM1, the CREB5 gene expression was decreased (Figure 6B). Overexpression of CREB5 genes by DNA transfection through electroporation induced cell apoptosis in SH-SY5Y cells. The enhancement of CREB5 expression exacerbated ROT-induced apoptosis and inhibited the anti-apoptotic functions of GM1 (Figures 6C, D).

## 4. Discussion

Intravenous infusion GM1 improved the motor and non-motor symptoms of PD patients. GM1 induced an obvious alteration of DNA methylation in peripheral blood. Hyper- and hypo-methylated genes were enriched in some pathways. The CREB5 gene were found to be involved in dopaminergic synapses. GM1 treatment might affect the dopaminergic synapse to modify the progression of PD.

GM1 was one of the most prevalent types of gangliosides found in the brain. It had been shown to have anti-neurotoxic, neuroprotective, and neurotrophic activities both *in vitro* and *in vivo* (Chiricozzi et al., 2020). By inhibiting cytotoxicity and potentiating neurotrophic factors, GM1 changed the differentiation process, amplified responses to neurotrophic factors, and minimized acute nerve cell injury (Fazzari et al., 2022). Previous work demonstrated that short-term use of GM1 ganglioside resulted in significant symptom reduction in PD patients (Schneider et al., 2010) and GM1 application may have long-term benefit for PD patients (Schneider et al., 2013). These observations revealed that GM1 therapy, in the short term, ameliorated the symptoms of PD patients and might provide some clinical benefits. In our results, GM1 use not only ameliorates the UPDRS III motor scale but also the non-motor symptoms such as cognitive impairment (MMSE scale), the quality**-**of**-**life (PDQ8) and sleep disturbances (PDSS-2 scale).

Previous research had shown that GM1 regulated active epigenetic modifications in the progression of Parkinson’s disease (Itokazu et al., 2017). In this paper, differential methylation was revealed in pre- and post-treatment of GM1. We also use the correlation analysis to explore the relationship between UPDRS III and GMP levels. There are 7 of DMP groups correlated with motor symptoms. The results suggested that GM1 treatment might modify the pathology of PD through DNA methylation.

Genes with differential DNA methylation points between pre- and post-treatment with GM1 were enriched in some pathways. In the top 10 pathways, Regulation of actin cytoskeleton, MAPK signaling pathway, Chemical carcinogenesis - receptor activation, GABAergic synapse, Morphine addiction, AMPK signaling pathway, Dopaminergic synapse, were correlated with the pathogenesis of PD. The CREB5/CACNA1B/GNB4/PPP2R5A gene was found to be closely related to Parkinson’s disease in pathways of dopaminergic synapses. Furthermore, in ROT-based cell models, the significant alteration of CREB5 gene expression was also involved in the treatment of GM1. In contrast with the ROT group, GM1 administration decreased cell apoptosis and the gene expression of CREB5. Treatment with GM1 at a proper concentration decreased ROT-induced apoptosis, but high- or low-concentration GM1 applications had no effect on cell apoptosis. The results indicated that 80 μM GM1 was the optimal concentration and exerted major protective effects on cell apoptosis in ROT-induced cell models.

The CREB5 gene encodes a cAMP-responsive element binding protein that regulates the expression of dopaminergic neuron-related genes (Sharma and Singh, 2020). The role of CREB5 involved in dopamine (DA) receptor-mediated nuclear signaling and neuroplasticity (Wang et al., 2018). Therefore, GM1 treatment induced hypermethylation of CREB5 in clinical therapy and decreased CREB5 expression in ROT-based SH-SY5Y cell injury. Enhancement of CREB5 gene expression inhibited the anti-apoptosis function of GM1. As a result, GM1 treatment reduced cell apoptosis by inhibiting methylated CREB5 gene expression.

In summary, GM1 application was beneficial to the pathology of PD and relieved motor and non-motor symptoms through alteration of DNA methylation. Early use of GM1 might delay the progression of PD by epigenetic modifications.

## Data availability statement

The datasets presented in this study can be found in online repositories. The names of the repository/repositories and accession number(s) can be found in the article/Supplementary material.

## Ethics statement

The studies involving human participants were reviewed and approved by the Clinical Trial Ethics Committee of the Affiliated Hospital of Binzhou Medical College. The patients/participants provided their written informed consent to participate in this study.

## Author contributions

RW and ST: conceptualization, methodology, software, investigation, formal analysis, and writing—original draft. MW: data curation and writing—original draft. JZ: visualization and investigation. NW: investigation and supervision. FS: software and validation. JC: investigation and validation. HW and XZ: conceptualization, funding acquisition, resources, supervision, and writing—review and editing. All authors contributed to the article and approved the submitted version.

## Footnotes

1
https://www.ncbi.nlm.nih.gov/geo/query/acc.cgi?acc=GSE51921
