# Corrigendum: CREB5 hypermethylation involved in the ganglioside GM1 therapy of Parkinson's disease

**DOI:** 10.3389/fnagi.2023.1250440

**Published:** 2023-08-02

**Authors:** Rui Wang, Shanshan Tong, Mengdi Wang, Junjie Zou, Nan Wang, Fengjiao Sun, Xiaosheng Zhou, Jinbo Chen, Hongcai Wang

**Affiliations:** ^1^Department of Neurology, Binzhou Medical University Hospital, Binzhou, Shandong, China; ^2^Department of Neurology, Penglai People's Hospital, Yantai, China; ^3^Medical Research Center, Binzhou Medical University Hospital, Binzhou, Shandong, China

**Keywords:** monosialotetrahexosylganglioside (GM1), Parkinson's disease (PD), DNA methylation, rotenone (ROT), cell apoptosis

In the published article, there was an error in Figure 5A, Image d as published. We inadvertently used the wrong figure and need to correct the Figure 5A, Image d. The other elements of the Figure 5 remain the same. The corrected Figure 5A, Image d and its caption appear below.

**Figure 5:**
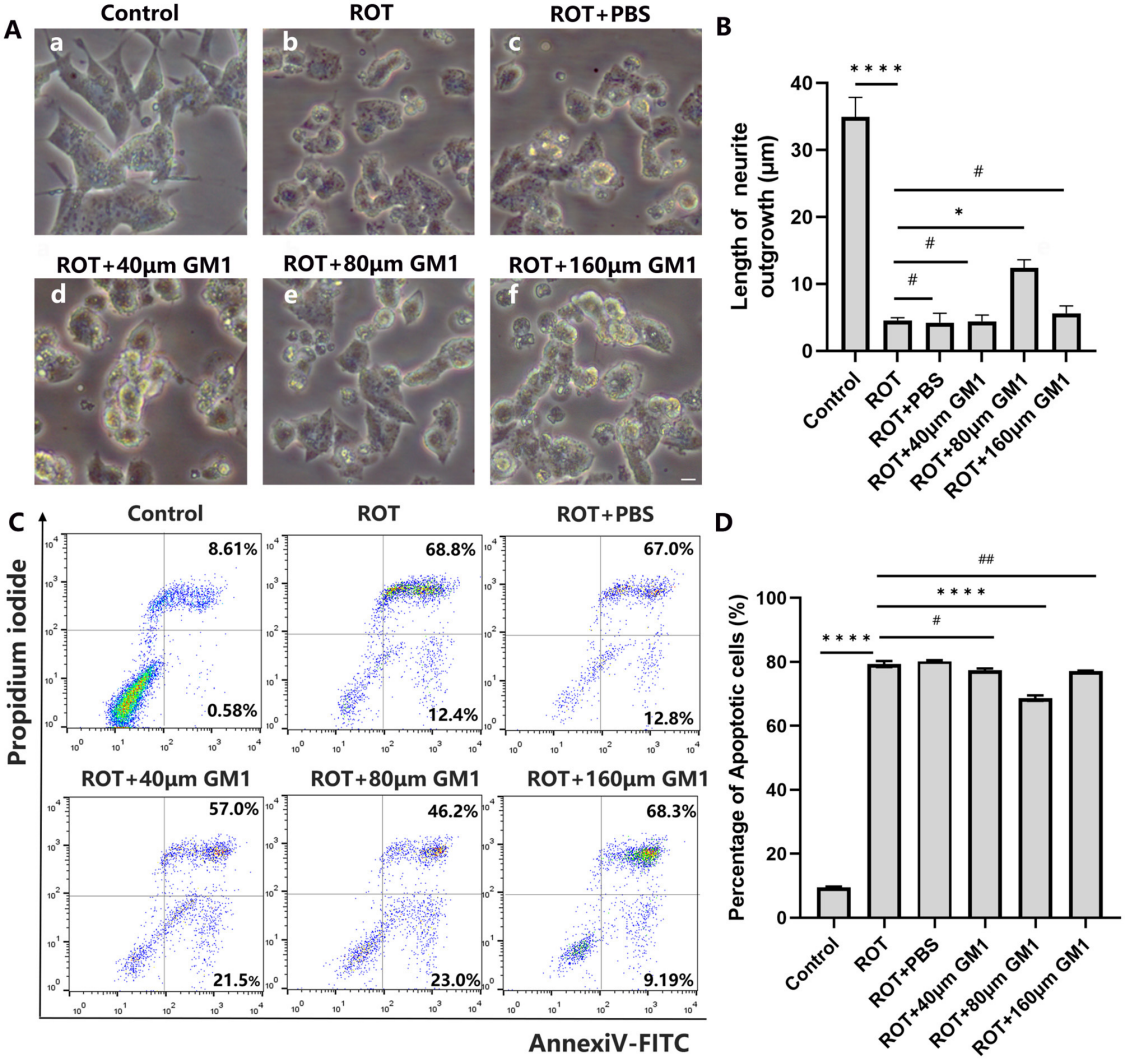
GM1 prevented ROT-induced cell apoptosis. **(A, B)** Impaired neurite outgrowth was inhibited by GM1 addition. Scale bar = 10 μm. **(C, D)** GM1 decreased ROT-induced cell apoptosis. ^*^P < 0.05; ^****^P < 0.0001; ^#,##^*P* > 0.05.

The authors apologize for this error and state that this does not change the scientific conclusions of the article in any way. The original article has been updated.

