# Supplementary material for: CREB5 hypermethylation involved in the ganglioside GM1 therapy of Parkinson’s disease
Source: Front Aging Neurosci. 2023 May 31;15:1122647. doi: 10.3389/fnagi.2023.1122647 (PMC10264581; doi:10.3389/fnagi.2023.1122647)
Supplement: Supplementary file 2 [file Table_2.docx]

| **probe** | **Data** | **P** | **logFC** | **CHR_hg38** | **UCSC_RefGene_Name** |
| --- | --- | --- | --- | --- | --- |
| **cg11537619** | **iPSC** | **1.84E-02** | **-3.98E-02** | **chr8** | **PMP2** |
| **cg01170286** | **GWAS** |  |  | **chr11** | **DLG2** |
| **cg20369299** | **neuron** | **0.00359** | **-0.0711** | **chr2** |  |
| **cg11181458** | **GWAS** |  |  | **chr3** | **KPNA1** |
| **cg08464675** | **GWAS** |  |  | **chr16** | **CBFA2T3** |
| **cg03447556** | **neuron** | **0.00023** | **-0.09** | **chr3** | **XIRP1** |
| **cg03876085** | **GWAS** |  |  | **chr21** | **COL18A1** |
| **cg25638714** | **neuron** | **0.00022** | **-0.0855** | **chr8** | **ZC3H3** |
| **cg18129781** | **iPSC** | **6.03E-03** | **-8.15E-02** | **chr1** | **OTUD7B** |
| **cg12709692** | **neuron** | **0.000216** | **-0.128** | **chr4** | **KCTD8** |
| **cg27295342** | **neuron** | **0.000616** | **-0.0648** | **chr3** |  |
| **cg08331219** | **iPSC** | **2.99E-02** | **-2.72E-02** | **chr15** | **COPS2** |
| **cg07620838** | **neuron** | **0.00011** | **-0.122** | **chr6** | **FILIP1** |
| **cg08464177** | **neuron** | **0.000483** | **-0.15** | **chr8** | **BAI1** |
| **cg24736274** | **neuron** | **0.00106** | **-0.0576** | **chr6** |  |
| **cg14331619** | **neuron** | **0.00425** | **-0.0542** | **chr9** | **LOC158376** |
| **cg27303733** | **neuron** | **0.00236** | **-0.106** | **chr11** | **EIF3F** |
| **cg16950247** | **neuron** | **0.00237** | **-0.106** | **chr11** | **SLC22A24** |
| **cg13406243** | **GWAS** |  |  | **chr9** | **CACNA1B** |
| **cg12160233** | **iPSC** | **2.47E-02** | **-2.79E-02** | **chr10** |  |
| **cg19220607** | **neuron** | **0.000241** | **-0.143** | **chr19** | **APLP1** |
| **cg06135008** | **neuron** | **0.000468** | **-0.0698** | **chr13** |  |
| **cg08117918** | **iPSC** | **0.00237** | **-0.106** | **chr1** | **FLAD1** |

Supplement 2 The DNA methylation probes in the Gene Expression Omnibus (GEO) database and GWAS
